# Gender differences in trends of acute myocardial infarction events: The Northern Sweden MONICA study 1985 – 2004

**DOI:** 10.1186/1471-2261-8-17

**Published:** 2008-07-25

**Authors:** Dan Lundblad, Lars Holmgren, Jan-Håkan Jansson, Ulf Näslund, Mats Eliasson

**Affiliations:** 1 Department of Medicine, Sunderby Hospital, Luleå, Sweden; 2 Department of Community Health, County Council of Norrbotten, Luleå, Sweden; 3 Department of Medicine and Geriatrics, Skellefteå Hospital, Skellefteå, Sweden; 4 Cardiology, Heart Centre, University Hospital, Umeå, Sweden; 5 Department of Public Health and Clinical Medicine, University of Umeå, Umeå Sweden

## Abstract

**Background:**

The registration of non-fatal and fatal MI events initiated 1985 in the WHO MONICA project has been ongoing in northern Sweden since the end of the WHO project in 1995. The purpose of the present study was to analyze gender differences in first and recurrent events, case fatality and mortality in myocardial infarction (MI) in Northern Sweden during the 20-year period 1985 – 2004.

**Methods:**

Diagnosed MI events in subjects aged 25–64 years in the Counties of Norrbotten and Västerbotten were validated according to the MONICA protocol. The total number of events registered up to January 1, 2005 was 11,763: 9,387 in men and 2,376 in women.

**Results:**

The proportion of male/female events has decreased from 5.5:1 to 3:1. For males the reductions were 30% and 70% for first and recurrent MI, respectively, and for women 0% and 40% in the 55–64 year group. For both sexes a 50% reduction in 28-day case fatality was seen in the 25–64 year-group. Mortality was reduced by 69% and 45% in men and women, respectively.

**Conclusion:**

First and recurrent events of myocardial infarction was markedly reduced in men over the 20-year observation period, but for women the reduction was seen only for recurrent infarctions. Case fatality, on the other hand, was markedly reduced for both sexes. As a result of the positive effects on incidence and case fatality a substantial reduction was seen in total mortality, most pronounced for men.

## Background

Coronary heart disease (CHD) is the leading cause of death in Sweden as well as in most Western European countries and in the United States. Incidence and mortality rates in CHD have decreased substantially during the last decades, and this trend seems to continue [1-4].

At the start of the MONICA project in 1985 the incidence of, and mortality due to, myocardial infarction (MI) in the two northernmost counties in Sweden were the highest in the country. However, they have gradually approached the national average indicating a faster reduction in Northern Sweden than in the rest of the country [5]. Generally, in the Western world, the decreased mortality in CHD is due to a combination of declining incidence and improved survival. Better primary prevention and improvements in acute coronary care, including secondary prevention, may explain these impressing achievements [1].

Gender differences in both events and case-fatality, and thereby also in mortality due to CHD, have been shown in previous publications from northern Sweden and elsewhere. The downward trends in first acute MI, recurrent MI, case fatality and mortality have been particularly strong in men, whereas the declines in women have been less impressive [6-8].

The reasons behind the differing time trends after MI between the sexes have been much debated. Suggested explanations have included: problems for women to get a correct diagnosis due to more atypical clinical presentation and a resulting longer delay between onset and medical presence, later onset of the disease in life and less typical ECG changes during MI [7,9]. This gender discrepancy in MI trends makes long-term follow-up data even more interesting, since the results mirror changes in primary and secondary prevention in the population. Gender discrepancy also gives an indication of health care quality and whether care is delivered in an equitable manner.

Incidence and mortality due to CHD increase greatly with age, and thus a rapidly ageing population is a huge challenge for society. Factors affecting incidence and case fatality are of utmost importance for CHD mortality. Hence, time trends in incidence and case fatality in various age and gender groups are of profound interest and can assist public health investigators aiming to decrease cardiovascular morbidity and mortality. In this aspect, analysis of time trends based on standardized data-monitoring procedures is superior to analyses based on routine vital statistics. The MONICA centre in Northern Sweden has continued the MI registration after the WHO project was ended in 1995. Both first and recurrent MI events are analysed to understand the effects of primary and secondary prevention. Since out-of-hospital deaths are also included, the register accurately describes the total burden of the disease. We can now present time trends from 20 years of uniform registration with special emphasis on the effect of gender on first and recurrent events, case-fatality and mortality due to MI.

## Methods

The WHO MONICA project (Monitoring Trends and Determinants in Cardiovascular Disease) started in the beginning of 1980s. Data for MI and for stroke have been collected from 39 centres in 26 countries [10]. In Sweden two centres have participated, one in the south (Gothenburg area) and Västerbotten and Norrbotten, the two northernmost counties as one centre. The WHO MONICA project was ended 1995 but has proceeded as a local project in northern Sweden. Initially, subjects between 25 and 64 years of age were included. From 2000, patients aged 65–74 years were also included, but the results for that age group are not shown due to unreliable trends due to the short registration period starting 2000. The total number of MI events in subjects aged 25–64 years during the 20 years of registration was 11,763.

From 1985 to 2004 all hospital discharge records, general practitioners reports and death certificates with International Classification of Diseases (ICD) codes were screened for events. During the 20-year period, 1985–2004 the ICD classification was changed three times in Sweden. In the period 1985–86 ICD 8 was used (codes 410–413), from 1987–97 ICD 9 (codes 410–413) and thereafter ICD 10 (codes I20 – I24). For death certificates codes were 410–414, 798–799 (ICD 8 and 9) and I20–25, R96–99 (ICD 10) respectively. Strict WHO MONICA criteria have been used to validate medical history, clinical symptoms, electrocardiograms (ECG) and cardiac enzymes, in the same way for fatal as for non-fatal events as described in detail elsewhere [10-12]. Trained nurses, supervised by the register physicians, have registered the MI cases.

ECGs were examined using the Minnesota code. Originally, MI diagnoses were based on typical chest pain, cardiac enzymes and ECG findings. From the late 1990s troponins were introduced for diagnosis of MI, and since 2000 they are the biomarker used by all hospitals in Northern Sweden.

From 2000, MI diagnosis has been based on typical chest pain and biomarkers. If only one of these parameters were positive, ECG analysis was included to get the final diagnosis. Based on these parameters, survivors were diagnosed as a definite MI or non-MI. Subjects who died within 28 days from onset of MI were recorded as fatal cases. This includes prehospital deaths, patients who died in hospital and patients who were discharged alive, but died outside hospital within 28 days. An event was considered to be first ever for the patient if the patient's history was free from a previous clinically recognized MI, otherwise the event was considered as recurrent. Total events describes all events whether or not the patients have had a previous event. For fatal events both definite and possible infarctions were included. For this purpose information was also obtained from death certificates and necropsy reports when available [10]. In Sweden, official ischemic heart disease (IHD) mortality statistics are based on death certificates. Hence all death certificates were screened for acute IHD events. The frequency of necropsies were 56.4% among men and 54.1% among women. Case fatality is defined as the proportion of fatal events to all events during the 28 days following the MI.

### Statistical analyses

Continuous data are presented as mean and standard deviation (SD). The target population for men ranged in the area of 132 000 and 140 000 and for women 126 000 to 131 000 during the 20-year period. Event rates and total mortality are shown as 3-year rates except for 2003–2004 which was a 2-year interval. Case fatalities are shown as percentages for the same 3-year and 2-year intervals. Direct age standardization was used for total events and mortality. The numbers of cases were divided by the population in each age class and are presented as rates per 100.000 per year in each time interval described. Figures are age adjusted according to the age distribution of the total population 2003–2004 in the two northernmost counties.

### Ethical considerations

The Northern Sweden MONICA Study was approved by the Research Ethics Committee of Umeå University and the data handling procedures by the National Computer Data Inspection Board. Participants or relatives to non-survivors gave written consent.

## Results

Table 1 shows baseline characteristics of the subjects in the study. There were almost 4 times more male subjects than females. The number of total events in men declined from 1881 (1985–1987) to 827 (2003–2004). The decline for women was from 391 (1985–1987) to 248 (2003–2004). The proportion of female subjects with a first MI in relation to all MI was significantly higher than for male subjects (73.8 vs 67.0%, p < 0.001). The mean age at first MI did not change over the period. Women were approximately 1 year older for both first and recurrent MI. Case fatality within 28 days was slightly higher for men for both first and recurrent MI.

**Table 1:** Baseline characteristics of the study population.

		**MI, n (%)**	**Mean age, years (SD)**	**Died within 28 days, n, (%)**
**MALE**	First MI	6.292 (67.0)	55.4 (6.9)	1.448 (23.0)
	Recurrent MI	3.012 (32.1)	57.2 (6.2)	1.584 (52.6)
	Insufficient data	83 (0.9)	57.2 (6.8)	69 (83.1)
**All**		9.387	56.0 (6.8)	3.101

**FEMALE**	First MI	1.753 (73.8)	56.4 (6.9)	377 (21.5)
	Recurrent MI	608 (25.6)	58.1 (6.2)	307 (50.5)
	Insufficient data	15 (0.6)	57.1 (6.8)	11 (73.3)
**All**		2.376	56.8 (6.8)	695

SD = standard deviation, n = number

**Figure 1:**
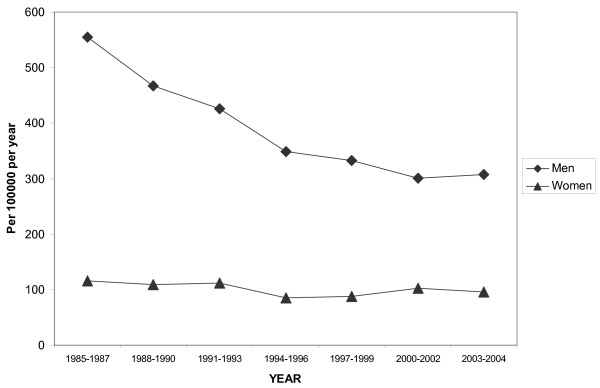
Age-standardized total event trends in MI for men and women aged 25–64 years.

From 1985 to 2000–2002, the total MI events in men aged 25–64 years decreased from 555 to 300/100,000, a 45% reduction. No further decrease was seen in 2003–2004 as compared with 2000–2002 (Figure 1). In women total events were 100/100,000, without any decrease since 1985. This has lead to a reduced risk gap between men and women from 5.5:1 in the period 1985–87 to 3:1 in the period 2003–2004.

**Figure 2:**
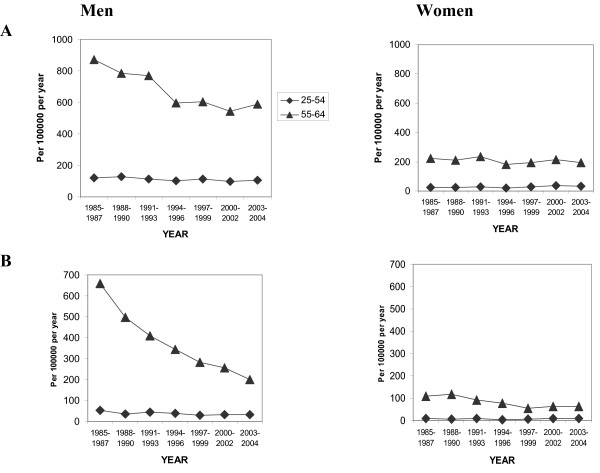
Time trends in A) first and B) recurrent MI for men and women in different age groups.

Data on events in two age groups and according to first or recurrent MI are given in Figure 2. The changes for men were pronounced. The reductions in first and recurrent events were 30% and 70%, respectively in the 55–64 year age group. For first MI the same trend was seen as in Figure 1 with the major decline occurring between 1985 and 1994–96. In contrast, the declining trend in recurrent MI continued over the study period. There was no obvious decline in the events for the youngest age group. For women, there were no changes in first MI events in any age group, but women aged 55–64 showed a 40% decrease in recurrent MI. In the younger group (25–54 years) the event rate was low and no change over time was noted.

**Figure 3:**
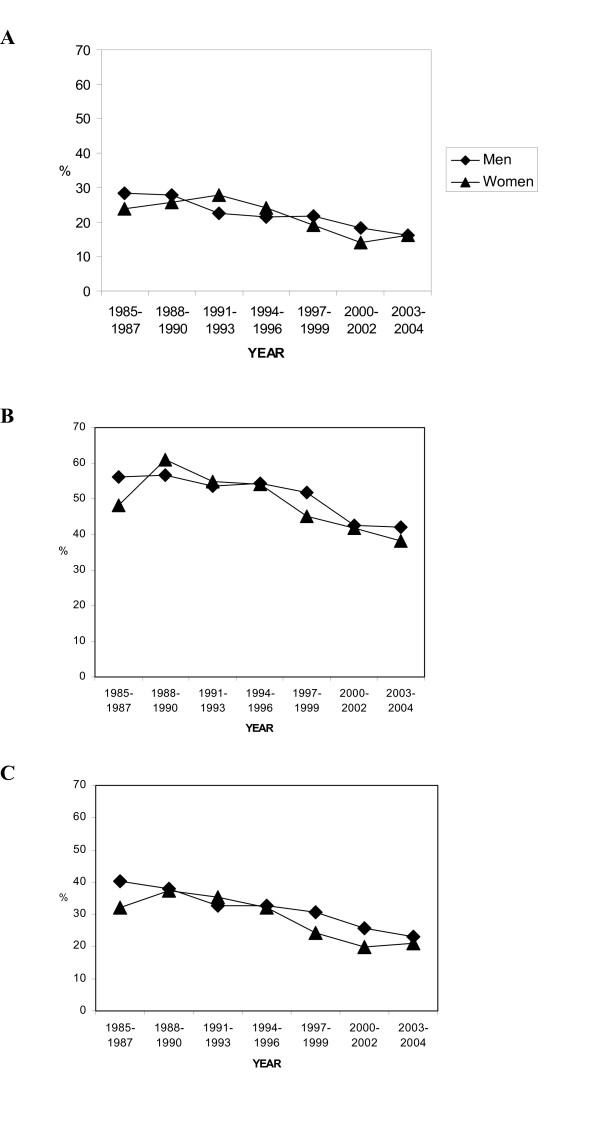
**Case-fatality trends for men and women aged 25–64 years.** A) first MI, B) recurrent MI and C) total events.

No obvious difference between the sexes in 28-day case fatality was seen (Figure 3). Over time, case fatality in first MI declined by almost 50% for both sexes. The reduction in recurrent MI was around 30% for both sexes, but remained three times higher than first MI over the study period. Both sexes showed an almost 50% total reduction in case fatality in all MI.

**Figure 4:**
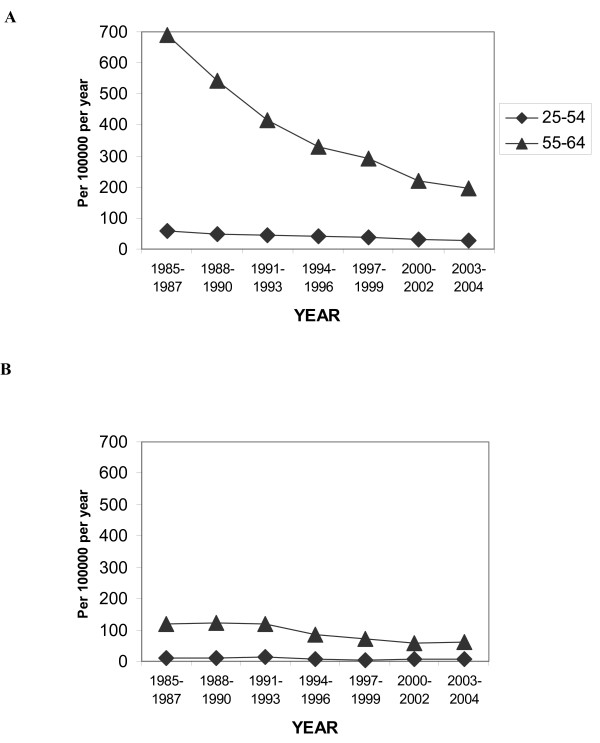
Mortality time trends in MI for A) men and B) women in different age groups.

**Figure 5:**
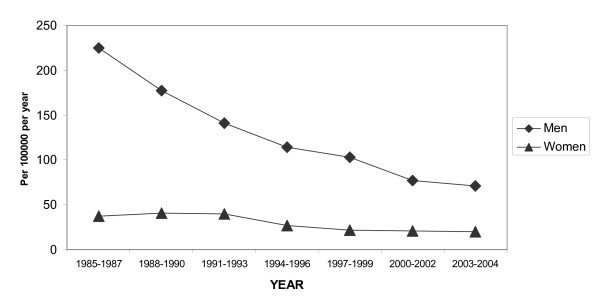
Age-standardized mortality (fatal definite + fatal possible) time trends in MI for men and women aged 25–64 years.

Mortality has been reduced since 1985 by 72% for 55–64 year old men but less (50% reduction) for women in the same age group (Figure 4). For men in the youngest age groups the decline was 50%, but for women of comparable age the numbers are too small and not conclusive. Mortality within 28 days for all MI has been reduced by 69% and 45%, in men and women, respectively (Figure 5).

## Discussion

The Northern Sweden MONICA study showed declining trends in MI events and mortality over 20 years, and to our knowledge, this is the longest population-based study published relying on standardized data collection procedures. The period 1997–2004 was included in addition to previous publications and hence data from recent years are available. Among women the events are declining mainly for recurrent MI, whereas both first and recurrent MIs decline among men with the best result for recurrent MI. Case-fatality trends are similar for both sexes with a 50% improvement from 1985 to 2004. The combination of lower incidence and higher survival rate translates into a substantial improvement in mortality, most pronounced in men. Thus, we can extend and confirm previously published data from Northern Sweden [8].

Comparisons with results from other studies should be done with caution due to possible methodological differences. However, other MONICA centres have published trends in MI events and mortality and, because of the common MONICA standards used, these studies are more easily compared. The time span differs, as most trend studies ended around the mid 1990s. In addition, treatment principles change continuously, thus making a comparison with our study not entirely easy.

In the Finnish FINAMI study, incidence of recurrent MI events was markedly reduced and first MI events were substantially reduced between 1983 and 1997 among men and women aged 35–64 years [13]. Similar results were found for both sexes. This differs considerably from our results in comparable age groups where the reduction for a first MI in women was lacking and the reduction for recurrent MI in women was lower and only found among those 55–64 years old. For men the reduction in recurrent MI in our study was similar to the Finnish results, but for first MI the Swedish reduction was less. The case fatality trend was markedly better for men in our study and on the same level for women. Mortality reduction was slightly better for men and was slightly worse for women in our study.

The MONICA-Catalonia study 1983 to 1997 [14] showed an increase in MI events for men but a decreased case fatality. Case fatality for women was higher than in Northern Sweden, and no decrease was seen over the time period. The authors suggest gender disparities in hospital care as an explanation. Both these results are in contrast to our data.

Changes in the classic risk factors seem to partly explain the variation in population trends in CHD [15]. The burden of cardiovascular risk factors in northern Sweden has changed substantially during the last 20 years as shown in the population surveys initiated in 1986 as a part of the MONICA study. At five different time points from 1986 to 2004 randomly chosen inhabitants in the age groups 25–74 years old (1986 and 1990 only 25–64 years old) were invited to a population survey where blood pressure, cholesterol level, smoking habits, body mass index (BMI), waist circumference, diabetes and glucose tolerance, physical activity and social factors among others were examined.

Survey data show that smoking has decreased in the population. In fact, the prevalence of male smokers in northern Sweden was lowest among the WHO MONICA populations at the start and has decreased further since then. In contrast, the prevalence of female smokers is higher than for men which is unique in Western Europe [16,17]. The use of moist oral snuff, "snus", is popular among men in Sweden. Its use has increased substantially among men and increased modestly among women. The possible effect of snuff on CHD has been debated, but recent studies support many previous ones showing no increase in the risk of MI [18,19].

Total cholesterol levels have decreased considerably during the study period but men still exhibit slightly higher levels than women, and lipid-lowering drugs have contributed only marginally [20]. Blood pressure has decreased, but only recently, with no major differences between the sexes [21]. Obesity and overweight are less prevalent in Sweden than in many other countries and in the USA. Nevertheless, the proportion of obese people has doubled and the proportion of overweight men has increased by about 30%, women by about 20%, since 1980 [22]. Because the increased weight is accompanied by decreased cholesterol levels, a high caloric intake in combination with less intake of saturated fats must lie behind these time trends. In fact, self reported reduced consumption of saturated fats has also been shown [23]. Physical activity has increased slightly since the first survey with no difference between the sexes.

Diabetes is a strong risk factor for CHD and is more common among men than women in northern Sweden. The decline in incidence and mortality due to coronary artery disease found in non-diabetics was not seen in diabetics as shown in a recent publication [24]. This indicates that diabetics have not benefited from intensified cardiovascular prevention and treatment to the same degree as patients without diabetes. However, there were no indications of any gender difference.

In all, the impact of changes in risk factors on CHD is complex. Also, during this period of major risk factor changes, treatment with thrombolytics and secondary prevention with medication such as aspirin, beta blockers and ACE-inhibitors have been introduced. Invasive treatment with percutaneos coronary interventions and coronary bypass operations have increased significantly. These changes in coronary care and secondary prevention have contributed significantly to the decrease in morbidity and mortality [25].

Risk factors are generally lower in the female population in Sweden with one important exception. Smoking is more common among women than men, and it is declining at a lower pace in women. This may contribute to the observed result where no decrease in the incidence of first MI is seen among women. Thus, a thorough analysis on the handling of risk factors and primary prevention in women, both in the population at large and through primary health care, is called for. For men, the positive trend in first MI correlates fairly well with the decrease in smoking and lower cholesterol levels previously observed in northern Sweden.

In the present study, recurrent MI in all but the youngest age groups among women showed an ongoing declining trend similar to the positive trend for men. Interestingly, the risk for reinfarction decreased already during the late 1980s for men but not until the mid 1990s for women. The same trends were seen for total mortality. This indicates a time lag in implementing adequate medical care and secondary prevention for women. This should be considered when novel medical treatments are introduced in order to ascertain equal treatment between the sexes. The positive trend seen in the last decade shows that cardiovascular treatment now has been equalized between the sexes.

Gender differences in the clinical manifestation and prehospital delay of MI have been suggested. Therefore the Swedish media has asked whether women with MI are mistreated. However, in northern Sweden we found no major gender differences in type of symptoms or time between symptom onset and medical presence as defined in the WHO MONICA protocol [26].

A limitation of the present study is the introduction of troponins as markers of myocardial injury, hence markers not included in the basic MONICA criteria for validation and classification of events. This means that MI definitions before and after the year of 2000 differs somewhat. It is however difficult to handle this situation when all centres in Sweden changed their diagnostic markers and at the same time stopped using markers decribed in the WHO MONICA project, and it is also virtually impossible to include exactly the same definition for MI in all participating centres throughout a study period of more than 20 years.

Troponins are more sensitive than previously used enzyme markers, and hence more patients are diagnosed as having an MI [27-32]. The introduction of troponins could explain the result with no further reduction in incidence for men 2003–2004 as compared with 2000–2002. MI without ST elevation (NSTEMI) has been suggested to be more frequent for women [7,33-35]. The more frequent NSTEMI in women, together with the higher sensitivity of troponin, may have contributed to the higher number of MI, hence less reduction in first events and a lower case fatality for women in the registry since 2000. Thus the observed effects of troponins may be false negative with respect to the observed events and the impact of this more sensitive marker on MI infarctions will be interesting to follow in future analysis.

The validity of our findings is strengthened by the strict and uniform use of the MONICA criteria over the whole period. The use of three different ICD classification systems during the 20-year period should not be a bias since the codes only have been used to collect possible cases. For diagnosis the same MONICA definitions have been used during the period. A major drawback of our registry is the upper age limit chosen in the WHO MONICA project since the majority of female MIs occur in older women. However, future analyses will include at least the 65–74 year age group starting in 2000.

## Conclusion

Between 1985 and 2004, the incidence of MI decreased for men with a substantial decline both in first and recurrent MI, whereas the incidence declined mainly for recurrent MI among women. This suggests an inferior effect of primary prevention in women. Case fatality was reduced by half in both men and women, indicating a similar and positive effect of acute coronary care. As a result of the positive effects on incidence and case-fatality, substantial improvements were also seen in total mortality, most pronounced in men.

## Competing interests

The authors declare that they have no competing interests.

## Authors' contributions

DL participated in the design of the study, performed parts of the statistical analyses and drafted the manuscript. LH performed the major part of the statistical analyses. J-HJ, UN and ME participated in the design of the study and helped to draft the manuscript. All authors read and approved the final manuscript.

## Pre-publication history

The pre-publication history for this paper can be accessed here:



## References

[B1] Tunstall-Pedoe H, Kuulasmaa K, Mahonen M, Tolonen H, Ruokokoski E, Amouyel P (1999). Contribution of trends in survival and coronary-event rates to changes in coronary heart disease mortality: 10-year results from 37 WHO MONICA project populations. Monitoring trends and determinants in cardiovascular disease. Lancet.

[B2] Rosen M, Alfredsson L, Hammar N, Kahan T, Spetz CL, Ysberg AS (2000). Attack rate, mortality and case fatality for acute myocardial infarction in Sweden during 1987–95. Results from the national AMI register in Sweden. J Intern Med.

[B3] McGovern PG, Jacobs DR, Shahar E, Arnett DK, Folsom AR, Blackburn H, Luepker RV (2001). Trends in acute coronary heart disease mortality, morbidity, and medical care from 1985 through 1997: the Minnesota heart survey. Circulation.

[B4] Abildstrom SZ, Rasmussen S, Rosen M, Madsen M (2003). Trends in incidence and case fatality rates of acute myocardial infarction in Denmark and Sweden. Heart.

[B5] (2005). Myocardial infarctions in Sweden 1987–2002.

[B6] Peltonen M, Lundberg V, Huhtasaari F, Asplund K (2000). Marked improvement in survival after acute myocardial infarction in middle-aged men but not in women. The Northern Sweden MONICA study 1985–94. J Intern Med.

[B7] Lundberg V, Wikstrom B, Bostrom S, Asplund K (2002). Exploring sex differences in case fatality in acute myocardial infarction or coronary death events in the northern Sweden MONICA Project. J Intern Med.

[B8] Messner T, Lundberg V, Bostrom S, Huhtasaari F, Wikstrom B (2003). Trends in event rates of first and recurrent, fatal and non-fatal acute myocardial infarction, and 28-day case fatality in the Northern Sweden MONICA area 1985–98. Scand J Public Health.

[B9] Lefler L, Bondy K (2004). Women's delay in seeking treatment with myocardial infarction. J Cardiovasc Nurs.

[B10] Tunstall-Pedoe H, Kuulasmaa K, Amouyel P, Arveiler D, Rajakangas AM, Pajak A (1994). Myocardial infarction and coronary deaths in the World Health Organization MONICA Project. Registration procedures, event rates, and case-fatality rates in 38 populations from 21 countries in four continents. Circulation.

[B11] Stegmayr B, Lundberg V, Asplund K (2003). The events registration and survey procedures in the Northern Sweden MONICA Project. Scand J Public Health Suppl.

[B12] Eriksson M, Stegmayr B, Lundberg V (2003). MONICA quality assessments. Scand J Public Health Suppl.

[B13] Salomaa V, Ketonen M, Koukkunen H, Immonen-Raiha P, Jerkkola T, Karja-Koskenkari P, Mahonen M, Niemela M, Kuulasmaa K, Palomaki P, Arstila M, Vuorenmaa T, Lehtonen A, Lehto S, Miettinen H, Torppa J, Tuomilehto J, Kesaniemi YA, Pyorala K (2003). Trends in coronary events in Finland during 1983–1997. The FINAMI study. Eur Heart J.

[B14] Sans S, Puigdefabregas A, Paluzie G, Monterde D, Balaguer-Vintro (2005). Increasing trends of acute myocardial infarction in Spain: the MONICA-Catalonia Study. Eur Heart J.

[B15] Kuulasmaa K, Tunstall-Pedoe H, Dobson A, Fortmann S, Sans S, Tolonen H, Evans A, Ferrario M, Tuomilehto J Estimation of contribution of changes in classic risk factors to trends in coronary-event rates across the WHO MONICA Project populations. Lancet.

[B16] Rodu B, Stegmayr B, Nasic S, Asplund K (2002). Impact of smokeless tobacco use on smoking in northern Sweden. J Intern Med.

[B17] Stegmayr B, Eliasson M, Rodu B (2005). The decline of smoking in northern Sweden. Scand J Public Health.

[B18] Hergens M-P, Ahlbom A, Andersson T, Pershagen G (2005). Swedish moist snuff and myocardial infarction among men. Epidemiology.

[B19] Wennberg P, Eliasson M, Hallmans G, Johansson L, Boman K, Jansson J-H (2007). The risk of myocardial infarction and sudden cardiac death amongst snuff users with or without a previous history of smoking. J Intern Med.

[B20] Eliasson M, Janlert U, Jansson JH, Stegmayr B (2006). Time trends in population cholesterol levels 1986–2004: influence of lipid-lowering drugs, obesity, smoking and educational level. The northern Sweden MONICA study. J Intern Med.

[B21] Jansson JH, Boman K, Messner T (2003). Trends in blood pressure, lipids, lipoproteins and glucose metabolism in the Northern Sweden MONICA project 1986–99. Scand J Public Health Suppl.

[B22] Bostrom G, Eliasson M (2006). Major public health problems – overweight and obesity. Scand J Public Health Suppl.

[B23] Krachler B, Eliasson MC, Johansson I, Hallmans G, Lindahl B (2005). Trends in food intakes in Swedish adults 1986–1999: findings from the Northern Sweden MONICA (Monitoring of Trends and Determinants in Cardiovascular Disease) Study. Public Health Nutr.

[B24] Rautio A, Lundberg V, Messner T, Nasic S, Stegmayr B, Eliasson M (2005). Favourable trends in the incidence and outcome of myocardial infarction in nondiabetic, but not in diabetic, subjects: findings from the MONICA myocardial infarction registry in northern Sweden in 1989–2000. J Intern Med.

[B25] Tunstall-Pedoe H, Vanuzzo D, Hobbs M, Mahonen M, Cepaitis Z, Kuulasmaa K, Keil U (2000). Estimation of contribution of changes in coronary care to improving survival, event rates, and coronary heart disease mortality across the WHO MONICA Project populations. Lancet.

[B26] Isaksson RM, Holmgren L, Lundblad D, Brulin C, Eliasson M Time trends in symptoms and prehospital delay time in women vs. men with myocardial infarction over a 15-year period. The Northern Sweden MONICA Study. Eur J Cardiovasc Nurs.

[B27] Koukkunen H, Penttila K, Kemppainen A, Penttila I, Halinen MO, Rantanen T, Pyorala K (2001). Differences in the diagnosis of myocardial infarction by troponin T compared with clinical and epidemiologic criteria. Am J Cardiol.

[B28] Meier MA, Al-Badr WH, Cooper JV, Kline-Rogers EM, Smith DE, Eagle KA, Mehta RH (2002). The new definition of myocardial infarction: diagnostic and prognostic implications in patients with acute coronary syndromes. Arch Intern Med.

[B29] Pell JP, Simpson E, Rodger JC, Finlayson A, Clark D, Anderson J, Pell AC (2003). Impact of changing diagnostic criteria on incidence, management, and outcome of acute myocardial infarction: retrospective cohort study. BMJ.

[B30] Salomaa V, Koukkunen H, Ketonen M, Immonen-Raiha P, Karja-Koskenkari P, Mustonen J, Lento S, Torppa J, Lehtonen A, Tuomilehto J, Kesaniemi YA, Pyorala K (2005). A new definition for myocardial infarction: what difference does it make?. Eur Heart J.

[B31] Abildstrom SZ, Rasmussen S, Madsen M (2005). Changes in hospitalization rate and mortality after acute myocardial infarction in Denmark after diagnostic criteria and methods changed. Eur Heart J.

[B32] Salomaa V, Ketonen M, Koukkunen H, Immonen-Raiha P, Lehtonen A, Torppa J, Kuulasmaa K, Kesaniemi YA, Pyorala K, FINAMI Study Group (2006). The effect of correcting for troponins on trends in coronary heart disease events in Finland during 1993–2002: the FINAMI study. Eur Heart J.

[B33] Paul SD, Eagle KA, Guidry U, DiSalvo TG, Villarreal-Levy G, Smith AJ, O'Donnell CJ, Mahjoub ZA, Muluk V, Newell JB, O'Gara PT (1995). Do gender-based differences in presentation and management influence predictors of hospitalization costs and length of stay after an acute myocardial infarction?. Am J Cardiol.

[B34] Tunstall-Pedoe H, Morrison C, Woodward M, Fitzpatrick B, Watt G (1996). Sex differences in myocardial infarction and coronary deaths in the Scottish MONICA population of Glasgow 1985 to 1991. Presentation, diagnosis, treatment, and 28-day case fatality of 3991 events in men and 1551 events in women. Circulation.

[B35] Hochman JS, Tamis JE, Thompson TD, Weaver WD, White HD, Werf F Van de, Aylward P, Topol EJ, Califf RM (1999). Sex, clinical presentation, and outcome in patients with acute coronary syndromes. Global use of strategies to open occluded coronary arteries in acute coronary syndromes IIb Investigators. N Engl J Med.

